# Priority-setting in public health research funding organisations: an exploratory qualitative study among five high-profile funders

**DOI:** 10.1186/s12961-018-0335-8

**Published:** 2018-06-22

**Authors:** Yuri Cartier, Maria I. Creatore, Steven J. Hoffman, Louise Potvin

**Affiliations:** 1 International Union for Health Promotion and Education, Saint-Maurice, France; 2 CIHR Institute of Population and Public Health, Canadian Institutes of Health Research, Toronto, Canada; 3 Dalla Lana School of Public Health, University of Toronto, Toronto, Canada; 4 Global Strategy Lab, Dahdaleh Institute for Global Health Research, Faculty of Health and Osgoode Hall Law School, York University, Ottawa, ON Canada; 5 McMaster Health Forum, Department of Health Research Methods, Evidence, and Impact, McMaster University, Hamilton, ON Canada; 6 Department of Global Health and Population, Harvard T.H. Chan School of Public Health, Harvard University, Boston, MA United States of America; 7 Institut de recherche en santé publique de l’Université de Montréal (IRSPUM), Pavillon 7101 avenue du Parc, C.P. 6128, Succ. Centre-Ville, Montréal, Québec H3C 3J7 Canada

**Keywords:** Public health research funding, Population health research funding, Priority-setting, Extramural research

## Abstract

**Background:**

Priority-driven funding streams for population and public health are an important part of the health research landscape and contribute to orienting future scholarship in the field. While research priorities are often made public through targeted calls for research, less is known about how research funding organisations arrive at said priorities. Our objective was to explore how public health research funding organisations develop priorities for strategic extramural research funding programmes.

**Methods:**

Content analysis of published academic and grey literature and key informant interviews for five public and private funders of public health research in the United Kingdom, Australia, the United States and France were performed.

**Results:**

We found important distinctions in how funding organisations processed potential research priorities through four non-sequential phases, namely idea generation, idea analysis, idea socialisation and idea selection. Funders generally involved the public health research community and public health decision-makers in idea generation and socialisation, but other groups of stakeholders (e.g. the public, advocacy organisations) were not as frequently included.

**Conclusions:**

Priority-setting for strategic funding programmes in public health research involves consultation mainly with researchers in the early phase of the process. There is an opportunity for greater breadth of participation and more transparency in priority-setting mechanisms for strategic funding programmes in population and public health research.

**Electronic supplementary material:**

The online version of this article (10.1186/s12961-018-0335-8) contains supplementary material, which is available to authorized users.

## Background

Population and public health (PPH) research is vital to improving the health of populations by advancing the knowledge of public health problems and solutions. Because of its interdisciplinary nature, PPH research may be funded through social sciences or even natural sciences research funding agencies, yet it is usually funded through health research funding agencies. In most high-income countries, health research funding represents a major investment for governments and philanthropic organisations. A recent study shows that, globally in 2013, the 10 largest health research funders invested a total of 37.1 billion USD [1]. To fund research, philanthropic and government agencies may operate either intramural programmes, wherein researchers employed by the organisation conduct research projects, or extramural programmes, to which eligible researchers, mostly but not exclusively from academic institutions, may apply. This paper examines how health research funding organisations make strategic decisions to influence research activity through extramural programmes.

Extramural research funding programmes are generally of two types, either investigator-driven or strategic. The former aims to be exclusively based on investigators’ creativity and their research ideas, whereas the latter proposes research topics based on strategic priorities to orient researchers’ requests for funding and overall research activity. The relevance of investigator-driven research proposals is gauged by peer-researchers with criteria that are mostly related to the project’s capacity to advance knowledge. Alternatively, in strategic research funding programmes, the relevance of a given research proposal to advance the research funding programme’s goals to impact people’s health is usually embedded in funding criteria. Both types of proposals are usually evaluated by peer researchers for quality of the proposal and feasibility. Although there is a dearth of data about the proportion of health research funds allocated to each of these types of programmes, it is understood that investigator-driven programmes tend to capture the majority of available research funds.

With regards to strategic funding, little has been published on the strategies and priorities set by health research funding organisations. The Public Health Innovation and Research in Europe project represents perhaps the most in-depth research on European public health research funders [2]. Additional studies have focused on the research funding priorities of individual funding organisations, like the National Institutes for Health Research (NIHR) [3, 4], the National Health and Medical Research Council (NHMRC) [5] and the United States National Institutes for Health [6]. Given that priority funding areas are identified and refreshed more or less frequently, the published literature, while historically interesting, does not offer insight into the current priority-driven funding trends. In addition, there is a lack of published literature on how these organisations make decisions on the priority-driven research they fund. This knowledge would be highly valuable to funders themselves, to benchmark or to learn from others. One recent exception is McGregor et al.’s [7] review of research priority-setting activities for 91 separate health research funding initiatives in low- and middle-income countries (LMICs). The review reported on the priority areas that resulted from these initiatives and provided categories for classifying them. In addition, they described the processes by which these areas were prioritised, organised into three categories, namely eliciting priorities, initial discussion and setting priorities.

This study’s objective is to explore how health research funding organisations in four different countries determine their public and population health research priorities in their strategic research programmes (in terms of priority areas and approaches), placed within the context of recent priorities.

## Methods

### Study design and population

This study is a cross-sectional qualitative survey of funding organisations’ priority-setting practices. It was commissioned by the Canadian Institutes of Health Research’s (CIHR’s) Institute of Population and Public Health in order to inform its own priority-setting exercise. Organisations in four countries (the United Kingdom, Australia, the United States of America and France) were selected. These countries were chosen on the basis of their comparability to Canada, with regards to commitment to funding extramural health research, presence of a centralised health research funding organisation with programmes for public health, being an OECD economy, and housing a research funding organisation in the top 10 globally, as is the case of CIHR (see Table one in [1]). The United States is an exception because its per capita investment in research is an order of magnitude higher than the other countries. We also prioritised funders with a comprehensive perspective on health research (from bench to population, etc.) as well as their accessibility (partners or members of the network of the International Union for Health Promotion and Education, strategic plans or other written documentation available in English or French).

Within these four countries, and with the help of a country-specific contact (see below), we selected a total of five organisations based on the following criteria: well-established (at least 10 years), national in scope, funding extramural PPH research through both investigator-driven and strategic funding competitions, and primarily funding research in their own country.

### Data collection

One seasoned PPH expert from each selected country was invited to a Country Contextualisation Committee. They provided information on their country’s most important health research funding organisations based on the above criteria. They were asked to prioritise organisations to be selected for this study, provide a list of key national health research orientation documents and reports, and identify potential key informants in the selected organisation(s), providing contact and introductions when appropriate.

In preparation for each interview with key informants, we thoroughly reviewed the websites and recent publications (from the past 5 years) of the selected organisations to extract relevant information on each organisation’s mode of operation, funding programmes, current research priorities and priority-setting mechanisms.

We approached key informants from each selected organisation. We gave informants an information form describing the conditions of participation in the study and invited them to participate. Informants provided written consent via email. Five interviews were conducted in April 2017. Before each interview, the informant was sent a summary of the information gathered about their organisation’s programmes, governance and priority-setting mechanisms in table format. Each key informant interview followed a pre-determined interview schedule, which was audio recorded and lasted 45–50 min. Key themes included the organisation’s governance structure, its strategic priorities, its PPH research priorities, and its decision-making groups and processes for determining which approaches and modalities to prioritise in funding PPH research. After the interview, recordings were paraphrased to generate a summarised transcript. Data from the transcript were added to the information from the initial tables.

### Data analysis

We generated case summaries of each organisation, synthesising data from both the key informant interviews and the document review (Additional file 1). These were sent to each informant so they could verify the accuracy and completeness of the information, and answer any follow-up questions that emerged from the synthesis process.

The research team met and, based on a preliminary analysis of the material, inductively produced a refined set of analytical categories. It was at this time that we schematised the process of determining research funding priorities in four non-sequential processes, including idea generation, idea analysis, idea socialisation and idea selection. Idea generation refers to how and by whom ideas that inform priority-setting are generated and gathered. Idea analysis is concerned with how and by whom collected ideas are processed and evaluated to gauge their importance to advance scientific knowledge. Idea socialisation relates to how and with whom selected ideas are discussed to assess their relevance for various stakeholders and knowledge users (including but not limited to the research community itself). Idea selection refers to who makes the final decision and how this person or group is bound by the whole process. We used these processes to then conduct cross-case synthesis.

This study received ethical approval from the University of Montreal’s Faculty of Medicine Ethical Review Board.

## Results

In order to fully understand how the different priority-setting mechanisms operate within the studied funders, we first explore the characteristics of each organisation as well as their most recent research priorities as of May 2017.

### Context

The five health research funding organisations studied represent different types of structures (Table 1). The NHMRC of Australia is a national organisation under the responsibility of the Minister for Health and the Minister for Sport, and is Australia’s largest health and medical research funder [8]. The NHMRC also provides public health guidance [9]. The United Kingdom’s NIHR is also public and is the research arm of the United Kingdom Department of Health [10]. It has several programmes, of which we took as our unit of analysis the Public Health Research Programme (PHRP), which exclusively funds extramural research studies [11]. In France, the Institut de recherche en santé publique (IReSP) is a consortium that brings together governmental research funding organisations and other research stakeholders [12]. It does not have an independent legal status and it is administered by its biggest flagship member, the Institut national de la santé et de la recherche médicale (INSERM) [13]. In addition to public research funding organisations, we selected two philanthropic foundations with a long history of health research funding. Based in the United Kingdom, the Wellcome Trust is a global charitable foundation with a primary focus on funding scientific research and research ecosystems [14]. In the United States, the Robert Wood Johnson Foundation (RWJF) is a nationally focused philanthropy that primarily funds programme and policy initiatives, but also invests a significant amount to fund research and evaluation [15].

**Table 1:** Contextual information on five public and population health research funding organisations, 2017

Category	NHMRC	IReSP	NIHR	Wellcome	RWJF
Type of organisation (foundation, agency, etc.)	National research funding and guidance organisation under the responsibility of the Minister for Health and the Minister for Sport	Public consortium of 26 health research funders and other research stakeholders; not incorporated as its own legal entity; hosted by national medical research institute	Public institute; research arm of the National Health Service	Global charitable foundation	National charitable foundation
Governance structure	NHMRC Council Principal Committees: Research Committee, Health Translation Advisory Committee, Health Innovation Advisory Committee, and the Australian Health Ethics Committee	Steering Committee (comité directeur) Director and deputy director Symbiotic governance: Aviesan (Alliance for life sciences and health) Public Health Institute has the responsibility for developing the overall public health research strategy and representing the public health research community. The same director leads both IReSP and Aviesan Public Health.	Senior Management Team of the Science, Research and Evidence Directorate at the Department of Health NIHR Advisory Board NIHR Strategy Board Each research programme has a director The NIHR PHRP has two boards – the Programme Advisory Board and Research Funding Board	Board of Governors Executive Leadership Team Expert Review Groups for thematic areas Each priority area has an Advisory Committee	Board of Trustees Executive Office Each signature research programme is run by a National Coordinating Center and has its own Advisory Board

*IReSP* Institut de recherche en santé publique, *NHMRC* National Health and Medical Research Council, *NIHR* National Institutes for Health Research, *PHRP* Public Health Research Programme, *RWJF* Robert Wood Johnson Foundation

Nearly all of the organisations have a vision or mission that includes improving the health of their respective nations. As a consortium, IReSP has a specific mandate to develop and promote public health research in France.

The amount of research funding expenditure varied among organisations, depending on the extent to which research funding was earmarked for PPH (Table 2). At the ‘open’ end of the spectrum we found NHMRC and Wellcome Trust, who have an overall research expenditure in the high hundreds of millions, mostly dedicated to investigator-initiated research. At the ‘specialised’ end of the spectrum, the IReSP exclusively funds priority-driven public and population health research and its research budget is 7€ million.

**Table 2:** Budget, extramural funding overview and priorities of five public and population research funding organisations, 2017

Category	NHMRC	IReSP	NIHR PHRP	Wellcome	RWJF
Annual research expenditure (estimated in USD)	619 million USD annually, with an additional 142 million USD over 5 years for dementia research	Approximately 7.6 million USD	NIHR as a whole: 306 million USD in research grants PHRP (our unit of study): 12.4 million USD	990 million USD	30 million USD
Types of science funded	Basic science, clinical medicine and science, public/population health and health services research	Population and public health research; health services research	Population and public health	Basic science, clinical medicine, population and public health, research ecosystems research, and health services research	Public and population health research, health services research
Schemes and programmes	Programme and project grants Individuals (fellowships) Centres of Research Excellence Infrastructure	Project grants Seed grants Network grants	Project grants	Individuals (fellowships) Team grants Seed grants Programme and project grants	Programme and project grants Research Hubs
Current priorities (can take the form of ‘priority areas’ or commissioned calls; can distinguish between issue-driven and process-driven priorities)	Institutional priority: The NHMRC has a cross-cutting priority to fund Aboriginal/Torres Strait Islander health research, with a target of at least 5% of research funding going to this area Much of the research funded in this area is population and public health related, though there is some in the other pillars Government-initiated priority: **-** Boosting Dementia, funding research into dementia across all four pillars for 5 years **-** Northern Australia Tropical Disease Collaborative Research Programme (5 million USD) In addition to these broad priorities, there are one-shot targeted calls for research that set aside a small part of the budget to address a pressing need for research As of May 2017, there were two TCRs open: • TCR into Dementia in Indigenous Australians • TCR into depression, anxiety and suicide among elderly Australians	Topic-specific calls are developed by one funding partner, whereas several funding partners contribute to the more general calls Taking the example of the 2017 Prevention CFP, the recurring priorities since the programme debuted are: 1. Cognitive research to support prevention interventions 2. Intervention research aiming to demonstrate the individual and collective effectiveness of interventions as well as to analyse their mechanisms of effectiveness and/or implementation There are also highlighted areas of special attention that change from year to year, and in 2017 they focused on: 1. Addictions 2. HPV vaccination 3. Measuring inequalities in health and the determinants thereof and evaluating the policies and interventions aiming to tackle said inequalities Investigators may submit proposals outside of these highlighted areas	The current overarching strategic priority of the programme is to increase the number of population intervention evaluations (relative to evaluations of individual/small group behavioural interventions) and system-level evaluations Current commissioned calls (issued 3–4 times per year): 1. Healthy diet in early years 2. Interventions in community organisations 3. Migrant health and wellbeing 4. Age-friendly environments 5. Better oral health 6. Interpersonal violence and abusive relationships in children and young people 7. Health and wellbeing for older employees in the workplace 8. Public mental health 9. Large scale public health studies	Priority areas 1. Diversity and inclusion 2. Drug-resistant infections 3. Our planet, our health 4. Research ecosystems in Africa and Asia 5. Science education 6. Vaccines Example: “Our planet, our health” endeavours to build an interdisciplinary research community around planetary health, create partnerships across sectors, inform decision-makers, and engage the public; 15 pilot projects have been funded since 2013, on top of 4 major interdisciplinary research partnerships focusing on global food systems and urbanisation	Four signature research programmes in 2017: 1. Evidence for Action 2. Health Data for Action 3. Policies for Action 4. Systems for Action

*IReSP* Institut de recherche en santé publique, *NHMRC* National Health and Medical Research Council, *NIHR* National Institutes for Health Research, *PHRP* Public Health Research Programme, *RWJF* Robert Wood Johnson Foundation, *TCR* targeted call for research

Aside from the IReSP, nearly all funders dedicated a majority of their research budgets to investigator-initiated research. The larger entities (NHMRC, Wellcome, NIHR as a whole) also have a variety of schemes to fund people and infrastructure.

### Priorities

We identified priorities operating on two levels, namely broad macro-level priorities and operational, priority-driven funding schemes (Table 2). Macro-level priorities highlight the direction in which the funder aims to orient the research field and the kinds of science that best serve the organisation’s mission and/or country’s health needs. For example, the NHMRC has an agency-wide priority to fund Aboriginal/Torres Strait Islander health research, with a target of at least 5% of research funding going to this area. The RWJF built four Signature Research Programmes (Evidence for Action, Health Data for Action, Policy for Action and Systems for Action) to operationalise its broad level research priorities. Within these programmes, there are different modalities of funding, mixing both investigator-initiated research and a small amount of strategic funding. In addition, it has organisation-wide priority thematic areas as well as an overarching action framework for building what RWJF dub “*a culture of health*”, encompassing socioecological approaches and shifting cultural norms towards health equity. The three strategic priority configurations interact with each other, with programme outcomes informing research investment and vice versa, although it is unknown whether this happens in a systematic or ad hoc manner.

Priority-driven funding schemes included Targeted Calls for Research (TCRs) in the NHMRC, all requests for proposals in the IReSP, and Commissioned Calls in the NIHR PHRP. The Wellcome Trust’s Priority Areas differ slightly in this respect, as the calls for proposals within each area are for investigator-initiated research, but within the context of the organisation, we considered them to be priority-driven funding streams. The current priorities of these schemes across funders show a great diversity of health issues (dementia, addictions, mental health, oral health) and populations (though there is a shared focus on the elderly in NHMRC and NIHR PHRP). We found public health intervention research prioritised in three of the five participant organisations (IReSP, NIHR PHRP, RWJF). In the following section, we primarily focus our attention on the decision-making processes for priority-driven research, though, when appropriate, we do include those for broader priorities.

### Priority-setting mechanisms

#### Idea generation

For all studied funding organisations, researchers are involved in the process of generating ideas for future research priorities (Table 3). The mechanisms for their participation differ, wherein some are solicited through their membership on advisory committees/boards (NHMRC, NIHR, RWJF) while others are invited through targeted events, such as the ‘Frontiers’ meetings where Wellcome invite a pre-selected list of global experts on a specific topic (e.g. mental health). Both Wellcome and RWJF indicated having ongoing interactions and consultations with the research community, who can bring up gaps in research funding and suggest future priority areas. In France, the Aviesan Public Health Institute has the responsibility for developing the overall public health research strategy and representing the public health research community. This strategy feeds directly into that of the IReSP at the macro level. The two entities share a director and offices, which facilitates the flow of input.

**Table 3:** Priority-setting mechanisms of five public and population health research funding organisations, 2017

Phase	NHMRC	IReSP	NIHR PHRP	Wellcome	RWJF
Idea generation	Researchers Policy-makers Community/advocacy organisations	Researchers Potential funding partners Policy-makers (members of consortium)	Researchers Policy-makers Local decision-makers	Researchers Internal	Researchers Internal
Idea analysis	Internal scoping	Potential funding partners (members of consortium and external partners), internal scoping, and ad hoc external consultations	Internal scoping (NIHR)	Internal scoping	Internal scoping and consultation with other funders
Idea socialisation	Researchers on internal Research Committee	Potential funding partners	External stakeholder events	Researchers internal	Committees composed of researchers and policy-makers
Idea selection	Research Committee	Funding partners	Programme Advisory Board and Programme Director	Board of Governors	Advisory Board of research programmes; Board of Trustees

*IReSP* Institut de recherche en santé publique, *NHMRC* National Health and Medical Research Council, *NIHR* National Institutes for Health Research, *PHRP* Public Health Research Programme, *RWJF* Robert Wood Johnson Foundation

Public health policy-makers and other decision-makers were a second group involved in idea generation. This is the case for the NIHR PHRP; they convene stakeholder events to which they invite local and national policy-makers (e.g. public health directors).

In the IReSP consortium, funding partners and other consortium members constituted the main idea-generating stakeholders at the micro level, by suggesting or requesting requests for proposals through direct contact with the IReSP director.

As for community groups (NGOs, advocacy organisations), they were solicited to generate ideas for TCRs through an online portal in the NHMRC, but we did not find any instances of their formalised involvement in generating ideas among the other funding organisations, for example, through representation on an advisory committee.

In the case of RWJF, ideas for priorities could also come from within the organisation. For example, when staff from the ‘Healthy Children, Healthy Weight’ focus area department perceived a gap in policy research, they approached the Policy for Action research programme staff to propose a contribution of $500,000 for research on childhood healthy weight policies earmarked within the otherwise investigator-initiated 2017 call for proposals.

In addition to soliciting ideas from different groups of research stakeholders, ideas were generated through scoping reviews, portfolio analyses and other similar desk-based processes (NIHR PHRP). In the case of the IReSP, this scoping process had already been carried out by Aviesan in defining the national public health research priorities.

#### Idea analysis

Potential priorities are scoped through a variety of methods that can include portfolio analysis and review of government reports, guidance and the scientific literature (NIHR PHRP, Wellcome). Analyses are mainly performed internally within each organisation. As mentioned above, this scoping can be used to generate further ideas that are subsequently grouped (NIHR PHRP) or to refine the focus of a potential priority to maximise impact of funding (Wellcome). Assessment of ideas can follow a specific framework, as is the case of NHMRC, where a working committee prioritises ideas for TCRs according to pre-defined criteria in a publicly available framework [16, 17]. The committee analyses each proposal by scoring high, moderate or low on three criteria (disease status and research need, research translation, likely outcomes of funding the TCR) before being sent to the Research Committee.

#### Idea socialisation

Potential priorities are discussed to a greater or lesser extent with stakeholders before they are officially selected. As a research consortium, IReSP engages in a socialisation phase with its consortium members and other potential funding partners. Feedback is provided on an initial draft request for proposals and then partners are asked if they are willing to fund the idea. In other cases, socialisation may happen concurrently with idea generation at stakeholder events such as those organised by the NIHR PHRP and Wellcome.

Both foundations mentioned socialising their ideas among other research funders to ensure that the idea meets a need in the funding landscape and that it is complementary to the approaches and priorities of other funding organisations.

Additionally, groups charged with the selection process discuss ideas. NIHR PHRP’s Programme Advisory Board runs small group workshops at their meetings where they discuss ideas and score/rank up to 10 priorities. This is similar to the NHMRC working committee, which receives ideas for TCRs through its online portal and discusses them as part of their analysis phase described earlier.

We did not find any instance among the organisations or research programmes included in our study where potential priorities were socialised among the general public.

#### Idea selection

Priorities on the level of TCRs are often decided through an iterative process that involves more than one level of responsibility. For example, the NHMRC’s working committee makes a first-round selection before the Research Committee makes an assessment and selects those to be recommended to the CEO, with a recommended amount of funding. An Expert Group then drafts the specific text of the TCR, including its scope and modalities. The CEO formalises the recommendation through a decision. Similarly, the NIHR PHRP’s scoring and ranking exercise described above is followed by the programme team (secretariat) making the final decision on topics for commissioned research, in discussion with the PHRP Director, who has the final say. Once agreed, the secretariat prepares briefs for the commissioned calls and these then go to the Programme Advisory Board for comment and further prioritisation (in terms of timing of release). It is important to note that amounts for funding are not set for TCRs.

The responses of the private foundations highlighted their collaborative and iterative approach with the decision-making body. For example, in the Wellcome Trust, the Board of Governors plays an active role as a sounding board during the priority development phase, in addition to being responsible for the final decision.

The selection criteria for TCRs vary, and can include considerations of external factors, such as the larger research funding environment and the need for change, and internal factors, such as the organisation’s own funding history for the issue or its capacity to make an impact in terms of research capacity or health outcomes. Again, the IReSP consortium differentiates itself in that its selection is above all determined by the priorities of the different funding partners and members.

## Discussion

The five PPH research funding organisations under study varied in the areas, populations and methodologies they prioritised, as well as in the way these priorities were developed.

Organisations defined priorities at the macro level that interacted with prioritised approaches at the level of the call for proposals. We found a strong concentration of prioritisation of intervention research in some of the organisations we studied, and also a special consideration of priority populations. In some of the organisations, we found health equity as an end-goal to which research priorities were designed to contribute, whereas others focused on improving the overall health of the population.

We found that across the board, the public health research community was involved in generating ideas, but other groups of potential stakeholders had lesser involvement. The NIHR PHRP’s inclusion of public health decision-makers in both its stakeholder events and its decision-making committee is noteworthy in this regard, and a recent article found that the priority-driven research funded by the NIHR PHRP matched the areas that national guidelines recommended local authorities act on to improve population health [4].

We found that, within our small sample, potential priorities were not socialised in any forum that included the public or any groups that represent them such as community-based organisations or health-related advocacy organisations. The literature confirms that there are instances of this in clinical research funding areas [18], yet we were unable to find models for such consultation among PPH research funders. The James Lind Alliance in the United Kingdom, whose infrastructure is funded by the NIHR, is interesting in this respect, as it facilitates priority-setting partnerships between clinicians, patients and carers that discuss treatment uncertainties and develop jointly agreed lists of the top 10 research priorities for the condition or disease [19]. This clinical approach can yield many PPH research priorities, notably on effective interventions to prevent the condition, though it is interesting to note that it did not appear to play an explicit role in priority-setting in the NIHR PHRP. This kind of partnership may represent a missed opportunity that PPH research funding organisations could use and adapt with a wider range of potential stakeholders, to ensure that potential priorities not only meet the needs of research users but also are formulated in a way that helps and does not harm research users or beneficiary populations.

Some funders mentioned investigating ways to evaluate impact of their priority-driven research funding on outcomes related to health and wellbeing, and not just on standard academic measures such as numbers of publications, citations and trainees, but none currently do, with the possible exception of RWJF, which conducts 360° evaluation of its research programmes by interviewing diverse stakeholders. Some work is being done by RAND Europe and a team at King’s College (at the time of writing) to assess the impact of the NIHR PHRP’s commissioned research, from which the programme will start to examine how research should be prioritised in terms of its potential impact on health and health inequalities. This and other initiatives that explicitly build evaluation into an iterative priority-setting process represent promising developments that should be documented and studied.

The foundations included in this study made greater use of internal resources for the development of strategic funding priorities. Staff and board members were more involved in idea generation and selection than were observed in the public organisations. This may be due to the relative independence of foundations to determine their priorities, and in the case of RWJF, is related to the important links between the priorities of the research portfolio and those of its programmatic areas.

We have focused on a small number of PPH research programmes, agencies or foundations; however, there exists a much broader diversity of research funder and research prioritisation configurations within the countries where our selected funders operate [6, 20] as well as globally. The European Commission is one example of an alternative model for extramural research [21]. There are also WHO-led and other intergovernmental research priority-setting processes at regional and global levels to orient research investment of member states, with some literature examining these processes in detail in the LMIC context [7, 22]. Though outside the scope of the immediate investigation, they merit mention as vital parts of the health research funding ecosystem.

Studies in this ecosystem have examined who funds health research and how funding is allocated, for example, with respect to disease burden [23]. Within the domain of health research priority-setting, extant literature has focused primarily on global health or the LMIC context, and without necessarily distinguishing between processes instigated by research funders to inform their extramural research investments and those instigated by other stakeholders for other purposes. The Child Health Nutrition Research Initiative priority-setting process was one used in several LMIC contexts [24, 25]. This process, which involves the scoring of a list of hundreds of research ideas against criteria which are subsequently weighted by external stakeholders, may have some transferability to the high-income, domestic, extramural research funding schemes included in our study.

Three recent literature reviews offer complementary approaches to schematising and categorising health research priority-setting processes. McGregor et al. [7] found three phases very similar to ours, namely eliciting priorities, initial discussion and setting priorities. Another review by Bryant et al. [26], focused on health research funding in high-income countries, referred to ‘generating priorities’ to encompass all activities. They found that workshops, nominal groups and roundtables were commonly used to generate priorities, mirroring our own findings. Finally, Yoshida [27] reviewed the literature on priority-setting in health research globally to derive a list of the most commonly used methods.

Our qualitative study contributes to this body of review literature by combining an organisation-level (and sometimes programme-level) focus similar to that of Bryant et al. [26] with an analysis of the different phases of priority-setting processes like that of McGregor et al. [7]. This combined approach, as well as the narrow scope we defined, allowed us to discern interactions between organisational strategy and priorities defined at macro and micro levels.

Another contribution of our work is examining organisational decision-making processes, which are not well studied in the literature and which, as discovered during the course of this study, are rarely explained on websites or in corporate documentation. This lack of transparency was highlighted by Chalmers et al. [28] as one of the drivers of avoidable waste in research.

A checklist of health research priority-setting was published by Viergever et al. in 2010 [29]. The checklist offers general themes of the decisions that need to be made and steps considered to develop a priority-setting process for health research. Our work complements the checklist by providing examples within the PPH research funding sphere of which principles and methods leading funders are using to set priorities for strategic research funding schemes.

### Strengths and limitations

Our study used an expedited approach that allowed us to generate direct usable knowledge on priorities and priority-setting processes within a short timeframe. We were able to verify our findings both with the organisational informants and the country-level contacts. However, the small number of organisations and countries in our study are a ‘convenience’ sample and our results cannot be generalised to all funders of PPH research. Additionally, our unit of analysis varied depending on the way funding organisations were structured, which means that, in some cases, we compared units within organisations to entire organisations. This led to overlooking patient and public involvement in NIHR outside the NIHR PHRP [30] and we may be unaware of other such initiatives that were not uncovered during the course of our interactions with our informants. Similarly, our knowledge of scoping mechanisms is thin, limiting the extent of our analysis on how funders used the scientific literature to generate ideas, and more investigation would be warranted in this domain.

## Conclusions

Through this exploratory study, we showed there exist a variety of mechanisms through which PPH research organisations develop priority-driven funding streams. Researchers and public health decision-makers are the main social actors consulted with for generating, analysing and socialising ideas for priority, whereas final decisions are made internally. Future research could go further in analysing these organisational processes and their determinants (values, governance structures, etc.).

As alluded to earlier, given the lack of publicly available documentation on these processes, health research funding organisations should consider reporting how they develop priority-driven funding streams in corporate documents or on their websites. Further, funders should capitalise on opportunities to deepen participation of non-researcher stakeholders in generating and discussing ideas for priority-driven research funding streams, in step with calls for Responsible Research and Innovation [31].

In addition to funding organisations, these findings would also be valuable to researchers and research users, including policy-makers, community organisations and other stakeholders who wish to advocate for greater participation in the health research priority-setting process. Finally, other health research funding organisations and those outside health may consider the approaches and mechanisms explored within this study for potential transfer and adaptation to their own priority-setting practices.

## Additional file

Additional file 1: Appendix S1 Case: NHMRC (Australia). Appendix S2 Case: L’Institut de Recherche en Santé publique (IReSP). Appendix S3 Case: National Institutes for Health Research Public Health Research Programme (NIHR PHRP). Appendix S4 Case: Wellcome Trust. Appendix S5 Case: Robert Wood Johnson Foundation. (DOCX 35 kb)

## Abbreviations

CIHR: Canadian Institutes of Health Research
IReSP: Institut de recherche en santé publique
LMICs: low- and middle-income countries
NHMRC: National Health and Medical Research Council
NIHR: National Institutes for Health Research
PHRP: Public Health Research Programme
PPH: population and public health
RWJF: Robert Wood Johnson Foundation
TCR: targeted call for research
