# Supplementary material for: Priority-setting in public health research funding organisations: an exploratory qualitative study among five high-profile funders
Source: Health Res Policy Syst. 2018 Jun 22;16:53. doi: 10.1186/s12961-018-0335-8 (PMC6014000; doi:10.1186/s12961-018-0335-8)
Supplement: Supplementary file 1 — Appendix S1 Case: NHMRC (Australia). Appendix S2 Case: L’Institut de Recherche en Santé publique (IReSP). Appendix S3 Case: National Institutes for Health Research Public Health Research Programme (NIHR PHRP). Appendix S4 Case: Wellcome Trust. Appendix S5 Case: Robert Wood Johnson Foundation. (DOCX 35 kb) [file 12961_2018_335_MOESM1_ESM.docx]

Additional file 1: Appendices S1-S5

Appendix S1. Case: NHMRC (Australia)

The NHMRC is Australia’s largest health and medical research funder and also provides evidence based public health guidance. Its research budget, spanning its four pillars (basic science, clinical medicine and science, public/population health and health services research) and largely investigator-driven, is $840 million annually, with an additional $200 million over 5 years (2014-2019) for dementia research. Approximately 15% of research funding goes to population and public health (PPH), though the exact proportion varies from year to year depending on applications. NHMRC’s largest funding scheme is investigator-initiated Project Grants, open to any research question across its four pillars. It also manages a number of priority-driven programs. These programs can and do incorporate PPH research. For example, the Centres for Research Excellence allocate 4 CREs each year to the PPH stream (4 are allocated to HSR and 6 to clinical research).

The NHMRC is currently undergoing a full review of its grant schemes and the structure of its funding programs. As a result, there may be significant changes to these programs in the coming years. It is unlikely that the Centres for Research Excellence scheme will change.

The Australian Government has just established the Medical Research Future Fund (MRFF), a complementary fund to the NHMRC that is managed by the Department of Health. The NHMRC CEO is a member of the MRFF Advisory Board, working with the Australian Government to identify and fund priority areas in research according to the strategic priorities of the MRFF. The NHMRC may be involved in implementing MRFF measures, depending on requirements and how complementary those measures are with NHMRC’s current functions.

**Current priorities**

Institutional priority: The NHMRC has a cross-cutting priority to fund Aboriginal/Torres Strait Islander health research, with a target of at least 5% of research funding going to this area. Much of the research funded in this area is PPH related, though there is some in the other pillars.

Government priority: The Boosting Dementia initiative is a priority scheme, funding research into dementia across all four pillars for five years. The funding for this priority area is provided by the Australian government in addition to the NHMRC’s core budget. Another government-initiated priority area is the Northern Australia Tropical Disease Collaborative Research Program ($6.8 million).

In addition to these broad priorities, there are one-shot targeted calls for research that set aside a small part of the budget to address a pressing need for research. There are currently two TCRs open:

- Targeted Call for Research into Dementia in Indigenous Australians
- Targeted Call for Research into depression, anxiety and suicide among elderly Australians

**Priority setting**

There are four different mechanisms through which priorities can be determined in the NHMRC (for all research, not just in PPH):

1. Government priorities

The federal government can designate and allocate funding for its priorities, as is the case for the dementia and tropical disease priority areas.

1. Committees

The NHMRC has a number of Principal Committees: Research Committee, Health Translation Advisory Committee (HTAC), Health Innovation Advisory Committee (HIAC), and the Australian Health Ethics Committee (AHEC). These Committees report to the NHMRC’s Council, which is comprised of the heads of the Principal Committees. NHMRC also has a Principal Committee Indigenous Caucus (PCIC), involving the Indigenous representatives from the Council and each of the Principal Committees, and a Community and Consumer Advisory Group (CCAG). Members of Council and the Principal Committees are appointed by the Australian Minister for Health, on a part-time basis, for a period of up to three years. The Committees listed are operating in the current triennium (2015-2018).

The HTAC was brought up as being particularly important for PPH research priority setting. This committee’s aim is to “advise the CEO and Council of NHMRC on opportunities to improve health outcomes in areas including clinical care, public, population and environmental health, communicable diseases and prevention of illness through effective translation of research into health care and clinical practice”.

The HTAC’s activities are recorded in the Committee’s work plan, which is used to keep track of progress and to prioritize activities as needed at each meeting. The CEO, Council and Research Committee may recommend items to be discussed and prioritized at any meeting, in addition to what is listed in the Committee’s work plan. The Committee meets three to four times each year.

1. Partnerships

The NHMRC participates in a number of multilateral partnerships as well as bilateral agreements. The Global Alliance for Chronic Diseases is an example of a multilateral agreement that involves PPH research. When it comes to bilateral agreements, the usual model is that a discussion is held at a high level what kinds of research to focus on; then on a list of topic areas. After that, a workshop of researchers and policy makers from both countries is convened in order to refine the scope of what should be funded in the topic areas.

1. Targeted Calls for Research (TCRs)

There is a dedicated budget, decision framework ([link](https://www.nhmrc.gov.au/_files_nhmrc/file/grants/apply/targeted_calls/tcr_identification_and_prioritisation_framework_-_ceo_cleared_.pdf)) and process ([link](https://www.nhmrc.gov.au/_files_nhmrc/file/grants/apply/funding_rules/2017/identification_and_prioritisation_of_a_tcr_process_flowchart_-_ceo_cleared_12_feb_16_.pdf)) for targeted calls for research. TCRs must address a significant research knowledge gap or unmet need for which there is the potential to advance knowledge of the issue and/or link to government health or health research priorities. TCRs can come through various channels including: Council and the Principal Committees; Australian Health Ministers Advisory Council; or from community groups via the Targeted Call for Research Online Pathway.

The TCR Online Pathway ([link](https://www.nhmrc.gov.au/grants-funding/apply-funding/submission-targeted-calls-research-online-pathway-nhmrc-s-statement)) allows organisations such as not-for-profit bodies, non-government organisations and advocacy groups with an interest in public health to submit ideas for what kind of research should be funded using the TCR mechanism.

TCRs proposed through the first two channels are by definition high-priority and sent directly to the Research Committee. Those proposed through the third channel are assessed by an NHMRC working committee and prioritized according to pre-defined criteria (see Annex 1 of the decision framework) before being sent to the Research Committee. The Research Committee assesses the prioritized list biannually, deciding which proposals to recommend to the CEO for funding, with a recommended amount of funding. An Expert Group then drafts the specific text of the TCR, including scope and modalities.

All four of the aforementioned priority-setting mechanisms have resulted in PPH research related calls and projects.

**Exemplary program**

The Centres for Research Excellence is the only funding program currently in place that quarantines a proportion of its budget specifically for PPH research. 4 PPH CREs are allocated each year; each CRE is capped at $2.5 million over 5 years. In addition, there can be priority areas of research for which CREs applications are solicited (the priority areas for 2017 are Electro-magnetic Energy Research and Primary Health Care). The assessment criteria, with their emphasis on transferability and capacity-building, are considered a strong feature of the program:

1. Generate new knowledge that leads to improved health outcomes (20%)
2. Promote effective transfer of research outcomes into health policy and/or practice (20%)
3. Develop the health and medical research workforce by providing opportunities to advance the training of new researchers, particularly those with a capacity for independent research and future leadership roles (20%)
4. Facilitate collaboration (20%)
5. Record of research and translation achievement – relative to opportunity (20%)

Each year, the priority areas for CREs are decided by the CEO based on advice from the Principal Committees, government and policy partners.

Appendix S2. Case: L’Institut de Recherche en Santé publique (IReSP)

IReSP is a consortium of 26 health research funders and other research stakeholders. Its primary aim is to fund public health research, which it defines as “aiming to understand the influence that genetic, environmental and social determinants have on population health and to identify effective interventions to improve population health and reduce social inequalities in health” (Chêne and Alla 2015). Its research expenditure is around 7€ million each year, of which between 10-20% is intervention research.

IReSP is not incorporated as its own legal entity, but operates through its biggest member, Inserm. Its director and deputy director are university researchers whose institutions donate approximately 25% of their time to IReSP.

There is another national group called Aviesan (Alliance for life sciences and health) under which there are institutes on different thematic areas in health and science. The Aviesan Public Health Institute has the responsibility for developing the overall public health research strategy and representing the public health research community. While for other thematic areas the Aviesan institutes operated independently, for public health the IReSP predates Aviesan (established in 2007 and 2008, respectively) and therefore, the two entities have developed a symbiotic relationship. The same director leads both institutes; they share offices; they publish a joint newsletter; and they share staff.

The 26 members of the IReSP consortium vary in their type (agencies, institutes, foundations, universities) and role (funding partner or advisory). Other organizations may sponsor a call without becoming a member of the consortium. Funding partners can choose whether to allocate their contribution to the general pool or to quarantine their funds to specific calls or to specific projects.

**Current priorities**

All of its funding schemes are priority driven; some are topic-specific (autism, disability, etc.) and there are two general calls (health services research, prevention), within which specific priorities can be nested. Topic-specific calls are developed by one funding partner, whereas several funding partners contribute to the more general calls.

Taking the example of the 2017 Prevention CFP, the recurring priorities since the program debuted a couple years ago are: cognitive research to support prevention interventions, and intervention research aiming to demonstrate the individual and collective effectiveness of interventions as well as to analyze their mechanisms of effectiveness and/or implementation. There are also highlighted areas of special attention that change from year to year, and this year they focus on (1) addictions, (2) HPV vaccination, and (3) measuring inequalities in health and the determinants thereof and evaluating the policies and interventions aiming to tackle said inequalities. Investigators may submit proposals outside of these highlighted areas. The informant highlighted that combining the more open and targeted streams in one call was an advantage, in that it made it possible to meet the demands of both the national public health research strategy and the individual funders within one highly visible, cohesive call.

**Priority setting**

Priorities are identified at two levels:

- Macro level (Aviesan)

The President (of France) requested a couple years ago that all of the Aviesan institutes develop research priorities^[[1]](#footnote-1)^. In public health, this meant embarking on a formalized process involving a group of over 30 researchers, decision makers and practitioners. The group did intensive scoping of existing documents, priorities in other countries, policy and research guidance and built consensus on a list of four priorities:

- research in prevention, studying both determinants and interventions
- research on public policies and health
- research on health systems
- methodological research

The Governing Board (Comité Directeur) of IReSP approved the adoption of these priorities as their own.

- Micro level (IReSP)

There is an iterative process by which the IReSP (directors/staff) draw up a first draft proposing a CFP. In order to develop this first draft, they draw upon 1) Aviesan’s public health research priorities; 2) if applicable, the formalized proposals sent by funding partners (usually when there are targeted approaches, such as the DREES’s focus in 2017 on tackling social inequalities in health). Sometimes, the IReSP will develop a specific process for developing this draft, like convening an ad hoc committee. Once developed, the draft call is then sent to the main funding partners and can be discussed in one-on-one meetings. On the basis of these discussions, the draft is revised and a second version of the call sent to all potential funders within the consortium to see if they are willing to fund it and if there are any modifications they would like to make. Sometimes IReSP can proactively approach a non-member funder and ask them to join a CFP, as was recently the case with the national cancer institute INCa. Other times, the funder may approach IReSP with a desire to target funding to their priorities, and through discussion there may be a separate call or a quarantined substream in one of the general CFPs (as is the case with the addictions and HPV vaccination “highlighted areas” in the 2017 Prevention CFP). The latter is an action from the National Cancer Plan and is therefore a government-derived priority that is coordinated by INCa. Some funding partners only fund within their priority areas, while others pool their contributions to the general fund, and yet others will do both.

Appendix S3. Case: National Institutes for Health Research Public Health Research Programme (NIHR PHRP)

**Context**

The National Institute for Health Research in the UK is the research arm of the National Health Service (NHS) and is funded by the Department of Health in England. It contains a number of research programs that are primarily focused within the NHS, with the exception of the Public Health Research Programme (PHRP), which exclusively funds research on interventions outside the NHS. The program’s budget is £10 million (frozen since 2008), which is entirely dedicated to intervention research. While this encompasses the evaluation of both new^[[2]](#footnote-2)^ and existing interventions, the programme does not fund the development of interventions. Research that develops new interventions are funded through the MRC Public Health Intervention Development programme). The

The PHRP reimburses its director’s institution for 20% of his time.

The PHRP funds research through both researcher-led and commissioned calls. In both cases, proposals are first examined by the Programme Advisory Board (PAB), which is essentially composed of local and national public health policy makers. The PAB scores and ranks proposals based on their public health importance for the UK. Next, those with a sufficient score are reviewed by the Research Funding Board (RFB), which is composed of researchers. The RFB scientifically evaluates proposals and assigns a second score. Proposals scored highly in both groups are then reviewed by a Prioritization Group (PG) composed of chairs and deputy chairs of both the PAB and RFB. This group makes a final selection that takes both scores into consideration, as well as the budget, and submits this list to the Department of Health with a recommendation for funding.

**Priorities**

The current overarching strategic priority of the PHRP is to increase the number of population intervention evaluations (relative to evaluations of individual/small group behavioral interventions) and system-level evaluations.

The current commissioned calls, which are issued 3-4 times per year, cover the following topics: healthy diet in early years, interventions in community organizations, migrant health and wellbeing, age-friendly environments, better oral health, interpersonal violence and abusive relationships in children and young people, health and wellbeing for older employees in the workplace, public mental health, and large scale public health studies.

**Priority-setting**

Priorities are identified through a variety of groups and processes.

- Stakeholder events: the PHRP periodically convenes meetings where it engages with a wide group of public health stakeholders. These stakeholders are invited through professional associations (e.g. Association of Directors of Public Health) and other sectors (charities, etc.), as well as inviting expressions of interest from other interested parties. During these meetings, stakeholders identify the issues on which they feel evidence is lacking. For example, at a recent meeting, local public health officials asked if the NIHR could commission research that would guide them on what public health interventions they should *not* be investing in.
- Open channel: there is a public portal on the website that allows anyone to suggest topics for research. Few suggestions are made through this channel, most of which are from researchers.
- NIHR program team: There are staff members dedicated to scoping priorities for research. They synthesize information on research implications or recommendations for research from a variety of sources: the aforementioned stakeholder events, government reports, agency reports, NICE guidance, and all NIHR-commissioned research. This mass of recommendation is grouped into areas to generate a long list of potential priorities that is presented to the PAB.
- PAB: The PAB has the responsibility of prioritizing the long list of potential priorities. Small group workshops are run at PAB meetings, where they discuss and then score/rank up to 10 priorities. The work also involves clarifying the scope of priorities.
- PAB and RFB: Once a year the two groups meet together for a strategic meeting, which enables a discussion of priorities that combine perspectives of both policy makers and researchers. These meetings are for more general horizon scanning and discussion of upcoming priorities rather than decision-making.
- Gap analysis: The portfolio is regularly scrutinized every 4 months, before the next PAB meeting as part of the scoping process for topic identification. The team also does more in depth analysis periodically (once/year). This is done to see what the balance of funding is, and where there are gaps. However, there is a concern that focusing too heavily on gaps leads to commissioned calls that target niche topics while ignoring larger issues. There is ongoing discussion on how this process can become more objective, for example, by triangulating this analysis with considerations of morbidity and mortality.

The final decision on topics for commissioned research is made by the programme team (secretariat) in discussion with the PHRP Director, who has the final say. Once agreed, the secretariat prepares briefs for the commissioned calls and these then go to PAB for comment and further prioritization (in terms of timing of release).

Some work is being done by RAND Europe and a team at King’s College at the time of writing to assess the impact of commissioned research, from which the program will start to examine how research should be prioritized in terms of its potential impact on health and health inequalities.

Appendix S4. Case: Wellcome Trust

**Context**

The Wellcome Trust is a philanthropic organization founded in 1936 through the will of pharmaceutical entrepreneur Sir Henry Wellcome. It has a yearly research expenditure of around £800 million, of which the percentage allocated to PPH research is unknown. There is a predominant emphasis on researcher-led funding: across the entire foundation, £550 million is for researcher-initiated research (including fellowships) and £150 million for strategic programs. Within the division for population health, the proportion of researcher-led funding was even higher.

The Trust is overseen by a Board of Governors, while the Executive Team composed of its division leads and chaired by the Director, is responsible for day-to-day management and advises the Governors and the Director on strategic, planning, operational and policy matters. An Expert Review Group exists for many thematic areas (including one on PPH), and each priority area and funding scheme has its own Advisory Committee.

**Priorities**

In the past, targeted programs were focused principally on basic science and, though strategic, were not based on strategic priorities. This has changed with the most recent set of priority areas:

1. Diversity and inclusion
2. Drug-resistant infections
3. Our planet, our health
4. Research ecosystems in Africa and Asia
5. Science education
6. Vaccines

One current priority area has a particularly strong PPH focus. “Our planet, our health” endeavors to build an interdisciplinary research community around planetary health, create partnerships across sectors, inform decision-makers, and engage the public. 15 pilot projects have been funded since 2013, on top of 4 major interdisciplinary research partnerships focusing on global food systems and urbanisation.

Strategic priority programs are funded across Wellcome, not just in the science division.

**Priority setting**

Only recently has a formalized process been defined, which is linked to the fact that the strategic priorities are themselves new.

Wellcome staff are in constant contact with the scientific community, so many ideas are generated through dialogue with them as to what issues are particularly important to address. Mental health was initiated from the research community who felt that Wellcome (along with many other major UK funders) did not invest enough in mental health research. The department consequently decided to dedicate staff time to doing a scoping review. The scoping phase lasted approximately a year and a half, during which they reviewed Wellcome’s funding portfolio over the past 10 years related to mental health, consulted with external experts, convened a “Frontiers” meeting with 30 pre-selected mental health experts from around the world, and continued discussions internally. During this time, other departments undertook a similar process with other potential priorities. Ultimately, six ideas were pitched to a committee. This committee decided which ideas could move to the development phase. Mental health was selected by the committee and is now in development.

The official period of development involves assigning a team to the topic, potentially bringing in people from outside Wellcome to work on the team, and working during a set period of time with key milestones. The Board of Governors are involved throughout the development process as a sounding board to develop ideas. At the end, the team produces a formal application to the Board of Governors that presents the priority area, the projected activities, the expected impact, and the amount of funding that should be allocated. The Board of Governors examines the application and decides whether or not to invest in the area based on the following considerations:

- there’s a clear and compelling need for change
- [Wellcome’s] involvement will make a real difference
- [Wellcome will] develop an approach that's more than the sum of its parts by bringing together different strands of [its] existing work
- [Wellcome] can involve a range of partners or different perspectives
- [Wellcome] have a focused plan with clear targets.

Appendix S5. Case: Robert Wood Johnson Foundation

**Context**

The Robert Wood Johnson Foundation (RWJF) is a foundation whose overarching vision is to build a Culture of Health in the US. It currently focuses on funding program and policy initiatives in four main areas: healthy systems; healthy children, healthy weight; healthy communities; and health leadership.

RWJF funds research that aligns with their vision, without specifically defining population and public health research. Their research expenditure is about $30 million, none of which is etiological research. This budget does not include their other knowledge-related activities (county health rankings, developing indicator measures for community health and equity, tracking “sentinel communities” to measure changes in adopting a culture of health perspective, RWJF program evaluation).

**Priorities**

RWJF has four signature research programs:

1. Evidence for Action (E4A) launched May 2015
2. Health Data for Action (HD4A) launched April 2017
3. Policies for Action (P4A) launched January 2016
4. Systems for Action (S4A) launched November 2015

Each research program is managed by a National Coordinating Center housed outside of the RWJF. These centers serve both an operational and expertise role: they manage the application process and peer review; and they are selected on the basis of their expertise, which is used to orient the research program but which also can be used to advise RWJF on programmatic activities. E4A is fully investigator-initiated, while P4A and S4A have an investigator-initiated stream and a strategic stream allocated to ‘research hubs’ or ‘collaborating research centers’. The P4A program collaborates with 5 research hubs, which are funded to provide “a transdisciplinary setting to develop, implement, and manage a program of strategic policy research to explore how policies, laws and regulations in both the public and private sectors can support building a Culture of Health”^[[3]](#footnote-3)^. One example is the Center for Public Health Law Research at Temple University’s Beasley School of Law, which focuses on how laws and legal practices related to housing impact health and equity.

There is interaction between the four programmatic focus areas and the four signature research programs. For example, “Healthy Children, Healthy Weight” focus area staff approached the P4A research program with the idea of contributing $500,000 for research on childhood healthy weight policies earmarked within the otherwise investigator-initiated 2017 call for proposals, because they perceived a research gap in this area.

**Priority setting**

- Ideas can be surfaced by a senior staff member or board member. They will explore the landscape, the main players in this area and what they are doing. They fund someone to do a scoping review of the topic, and reflect on how the issue fits within current priorities. The informant gave the example of climate change: What is the health equity dimension? Are communities prepared for the more abiding (vs. acute) impacts of climate change? This overall discussion is followed by talks with the National Coordinating Center of the research program the issue would best be placed under.
- In other cases, the process can begin with the National Coordinating Center contacting RWJF with the idea, as an area in which research is lacking.
- Regardless of the genesis of the idea, the National Advisory Committees associated with each research program are responsible for formally vetting and discussing ideas to determine priorities.

Targeted CFPs

- At the research program’s launch, the first CFP is prone to be for investigator-initiated research. As time goes on, there may be gaps in the research that become evident, or a direction may emerge that the RWJF would like to go in. In these cases, a discussion takes place between the RWJF, the National Coordinating Center for the program, and if applicable the research hub associated with a specific area.
- Targeted CFPs may be put out only once or twice; they may be allocated a portion of the budget, with the rest left to investigator-initiated research.

**Exemplary program**

The Healthy Eating Research program ([link](http://healthyeatingresearch.org/)) “supports research on environmental and policy strategies with strong potential to promote healthy eating among children to prevent childhood obesity, especially among lower-income and racial and ethnic populations at highest risk for obesity”. It focus has shifted since its launch—at first the CFP were very broad, funding effectiveness studies for interventions in several settings. Over time the number of proposals increased, and the advisory group honed the focus of the CFP to areas where more research was still needed.

1. See report here: https://aviesan.fr/fr/aviesan/accueil/toute-l-actualite/plan-d-action-pour-l-organisation-de-la-programmation-de-la-recherche-en-sante [↑](#footnote-ref-1)
2. For example, the programme may fund studies that adapt or optimize interventions that have been developed in a different context or country. [↑](#footnote-ref-2)
3. See https://www.policiesforaction.org/research-hubs [↑](#footnote-ref-3)
